# Re-assessing the Foundations: Worldwide Smallpox Eradication, 1957–67

**DOI:** 10.1017/mdh.2019.77

**Published:** 2020-01

**Authors:** Sanjoy Bhattacharya, Carlos Eduardo D’Avila Pereira Campani

**Affiliations:** 1 Centre for Global Health Histories, Department of History, University of York, York YO10 5DD, UK; 2 Department of Health Sciences, University of York, York YO10 5DD, UK

**Keywords:** Smallpox Eradication, World Health Organization, World Health Assembly, International Health, Global Health, Variola

## Abstract

An expansive, worldwide smallpox eradication programme (SEP) was announced by the World Health Assembly in 1958, leading this decision-making body to instruct the World Health Organization Headquarters in Geneva to work with WHO regional offices to engage and draw in national governments to ensure success. Tabled by the Soviet Union’s representative and passed by a majority vote by member states, the announcement was subject to intense diplomatic negotiations. This led to the formation, expansion and reshaping of an ambitious and complex campaign that cut across continents and countries. This article examines these inter-twining international, regional and national processes, and challenges long-standing historiographical assumptions about the fight against smallpox only gathering strength from the mid-1960s onwards, after the start of a US-supported programme in western Africa. The evidence presented here suggests a far more complex picture. It shows that although the SEP’s structures grew slowly between 1958 and 1967, a worldwide eradication programme resulted from international negotiations made possible through gains during this period. Significant progress in limiting the incidence of smallpox sustained international collaboration, and justified the prolongation and expansion of activities. Indeed, all of this bore diplomatic and legal processes within the World Health Assembly and WHO that acted as the foundation of the so-called intensified phase of the SEP and the multi-faceted activities that led to the certification of smallpox eradication in 1980.

The World Health Assembly (WHA) of May 1980 hosted the celebrations of the worldwide eradication of naturally occurring smallpox. Caused by the variola virus, the disease had been striking different corners of the globe in regular epidemic cycles, and an international commission of experts met in Geneva in December 1979 to confirm its disappearance in nature. This certification had been justified with the help of data generated through an intensive, two-year long worldwide search for naturally occurring smallpox, after what turned out to be the last recorded case in 1977 inside Somalia.^1^ Celebrations of such successes are generally balanced on hagiographic references to the past and this event was no different. The achievement of smallpox eradication was linked to Edward Jenner’s 1796 discovery of an early version of a smallpox vaccine, even if there was extremely little in common between it and the great variety of vaccinal products, immunological understandings, epidemiological concepts, administrative innovations and societal interventions that had underpinned the worldwide fight against variola. The celebrations also began to manufacture exclusionary myths, built on the privileging of certain projects and their managers over the work of other actors. The resultant narratives generally ignored the longer histories of smallpox vaccination around the world throughout the twentieth century, as well as the influence of discussion, disagreement and accommodation between the World Health Organization’s Headquarters (WHO HQ) in Geneva, its regional offices and their member states in creating a multi-faceted programme.^2^ This article shows that it is more beneficial to adopt a different frame for the study of smallpox eradication.

## 1 Interrogating Multiple Historiographies

It would be fair to say that existing institutional histories, participant autobiographies and biographies, and academic studies of worldwide smallpox eradication have generally failed to provide us with nuanced assessments of the many organised efforts to improve and widen smallpox surveillance and vaccination work across Latin America and Asia during the 1950s and early 1960s. For example, official histories prepared or sponsored by the US Centers of Disease Control (CDC) propose a very distinctive historical and temporal frame, which argues that innovations by officials in western and central Africa, from the mid-1960s onwards, were central to the Smallpox Eradication Programme’s (SEP’s) worldwide success.^3^ This is a narrative describing how relatively small groups of US actors moved from one national context to another, all acting in seemingly uniform ways and spreading a core wisdom supposedly developed by a handful of visionaries that then acted as the driver for worldwide smallpox eradication.^4^ Herein, the embrace of these supposedly infallible precepts by national and local actors in developing countries, who usually only get fleeting mentions, becomes a civilisational marker. That is, evidence of the existence of scientific sensibilities and abilities among members of African and Asian national workforces, who CDC consultants were then able to marshal effectively and engineer the victory against smallpox.^5^ A grand diffusionist narrative, this is a tale of how a body of thought within a supposedly monolithic public health agency in the mid-1960s would go on to educate everyone else in the world between 1967 and 1977. It is noteworthy how little this formulation has shifted in narratives that involve ex-CDC actors, even as it has found new avenues of propagation in recent years. For example, the online Global Chronicles archival project is remarkable in its exclusivity in selecting interviewees from within and outside the CDC, even as it presumes to equate the subjective memories of the relative few with a ‘global’ perspective about an extremely complex international public health programme involving hundreds of thousands of workers from countries around the world.^6^ Such positivist narratives, highlighting the excellence and impact of the select few, continue to live through similarly exclusionary participant autobiographies that generally remain reliant on selective memory and nostalgia. Such work is rarely marked by self-criticality or humility, and is usually completely disengaged from detailed research with swathes of official correspondence available in WHO and government archives, which detail a range of strengths and frailties within all agencies involved in delivering the SEP.^7^

Even though the WHO HQ’s official history, *Smallpox and its Eradication*, published in 1988, is distinct from the CDC’s offerings, it has also been influential in presenting a very specific SEP timeline.^8^ This narrative privileges the work carried out between 1967 and 1977, presenting this so-called intensified phase of action as absolutely central to eradication. Therefore, the geographic regions where these activities were carried out receive maximum attention, and this goes hand in hand with the production of a constricted history of earlier efforts and the regions where these were delivered. Written by several SEP stalwarts, including Donald A. Henderson and Isao Arita, who headed the Smallpox Eradication Unit within the WHO HQ at different points of time, and Frank Fenner, who chaired the international eradication certification committee, its detail-heavy nature hides a number of ills. It is based on a selective use of a rich corpus of unpublished materials dealing with the SEP available in the WHO HQ’s archives.^9^ Few academic histories have openly recognised that this publication combines the authors’ own subjectivities with many intricate political negotiations within WHO frameworks and the governments of all countries described within it. Highlighting the roles played mainly by WHO representatives, this was, by no means, a comprehensive assessment of the great diversities of field-level experiments and innovations; it is largely a narrative about the top-down imposition of ideas for successful eradication. The book is, in essence, a diplomatic exercise that seeks to explain how finite sets of ideas allowed international and national actors, largely brought together by the WHO HQ, to expunge variola. That *Smallpox and its Eradication* does not present us with value-free sets of information on which there was wide-ranging agreement is proven by the wider body of work penned by its authors. For example, interpretative differences over timelines and the strategic worth of specific actors are clearly visible when one compares the biographies of Isao Arita and Donald Henderson. Both books refer to the work carried out across the world before 1967 only briefly. However, there are important terminological and analytical differences about what happened before the so-called intensification of the SEP. Arita recollects his experiences as a field epidemiologist in western Africa, where he refers to his visits to Mali, Nigeria, Afghanistan and Burma, and notes the existence of active national smallpox eradication initiatives. He labels these activities as the preparatory phase of the worldwide SEP.^10^ Henderson, by contrast, claims that the worldwide SEP struggled until US intervention from the mid-1960s, underpinned by President Lyndon Johnson’s political and financial commitments.^11^ There are other notable differences in the narratives as well. For example, there is a disjuncture between their descriptions of the attitude of Dr Marcolino Candau, the WHO Director-General (WHO DG) at the time the SEP was formally announced by the WHA in 1958 (and who remained in post till 1973). Arita highlights Candau’s defence of the goal of smallpox eradication in a WHO Executive Board (EB) meeting in 1966, describing a process where he fought for the strengthening of the SEP’s interlinked national chapters.^12^ For Henderson, this WHO DG was someone who opposed smallpox eradication, manoeuvring behind the scenes to derail the programme.^13^

Critical assessments of such interpretational dissonances in institutional and participant histories is substantially lacking in academic scholarship. This is perhaps explicable due to the Anglophone-centric research base of much of the available work, which is marked by a tendency to mainly ascribe value in relation to the creation of ideas and delivery of health programmes to English-speaking actors. However, scholarly biases also seem driven by factors other than the choice of lingua franca of communication of historical actors. This is visible in the stubborn tendency to avoid careful engagement with the papers and writings of prominent non-US and non-European actors, even when bodies of such work are made available in English. There is, for instance, a remarkable inability to consider Isao Arita’s published thoughts, retrospective or otherwise, even though he was the chief of the WHO HQ’s Smallpox Eradication Unit in the run-up to the formal certification of eradication (his biography being a good example). Such historical scholarship is often founded on received presumptions, where academics have generally focused on the opinion and actions of a handful of US participants, believing only that they were able to play meaningful international roles. Ideas of exclusivity in relation to SEP design and delivery are promoted by the unquestioning adoption of claims and timelines presented by representatives of institutions such as the CDC. Such historical work then emphasises the centrality of US influences throughout the SEP, starting with President Lyndon Johnson’s public announcement of support for a measles and smallpox vaccination programme in western and central Africa. Such an explanatory frame is visible in Bob Reinhardt’s book, where the WHA’s formal support in 1958 for smallpox eradication gets a fleeting mention, but there is no recognition or analysis of its almost immediate international impact (for example, in the shape of the influential WHA Resolution 12.54 passed in 1959).^14^ Such selectivity in research then appears to lead to the SEP’s intensified phase being described as an extension of the USA’s Cold War battlefields.^15^

More rounded and critical work on US engagements with the SEP is provided by Erez Manela. Here, retrospective imaginations of involvement in the programme are carefully unpicked and programmatic successes are attributed to ‘US funds and expertise with the Soviet capacity for vaccine production’.^16^ Anne-Emanuelle Birn uses a similar formulation, declaring that the combination of American epidemiological expertise and a Soviet vaccine were the key drivers of the SEP, even as she argues that smallpox presented an odd choice for eradication in 1966.^17^ Paul Greenough has given us an original and well-researched study of CDC officials working on smallpox epidemiology in East Pakistan in the late 1950s and early 1960s. However, this scholarship does not contain an assessment of how data collected from these initiatives connected to the work of WHO representatives given the responsibility for mobilising support for the newly launched SEP.^18^ An attendant historiographical narrative fuels the exclusionary approaches to the history of the SEP. A well-established body of scholarship has continued to favour what is best labelled a step-by-step approach to the study of international programmes involving the WHO, which is generally presumed to be equivalent to its Geneva-based HQ. This work promotes the argument that one campaign followed another, with minimal or no overlap; so, in such narratives, malaria eradication came first, followed by the fight against smallpox and that this was then succeeded by the advocacy of primary health care.^19^ This presentational device, in turn, manufactures constrained timeframes for each initiative, justifying their supposedly self-contained nature. All these narratives come together to provide us with a narrow understanding of the SEP, which is equated with the so-called intensified phase that was put into place in the latter half of the 1960s, after the completion of United States Agency for International Development (USAID) and CDC-supported work in western and central Africa. By highlighting the significance of the Cold War at the expense of other political movements and alliances, this body of work also combines to create silences about the extended history, great geographical spread and the significant impact of what Isao Arita has called the SEP’s preparatory phase in the 1950s and early 1960s.^20^

A body of more inclusive scholarship, which recognises the value of the voices of a wider range of actors in the worldwide SEP, also exists. This scholarship, generally speaking, examines how national political priorities influenced their own engagement with regional and international efforts. Paul Greenough’s path-breaking article about the use of force, and wide-ranging and frequently powerful nature of resistance in South Asia, pioneered work that consciously shifted away from narrow, heroic narratives of the programme.^21^ We have Gilberto Hochman’s fine-grained research about Brazil, which details how the national authorities ignored smallpox during the fifties even though the WHO Pan American Health Organization (PAHO) had launched a continental eradication effort in 1950. According to Hochman, the situation changed during the sixties, following a chain of events that made the Brazilian central government more interested in international partnerships. Hochman reveals how some national representatives took advantage of the new political developments in Latin America, especially the work of Juscelino Kubitschek with the so-called Alliance for Progress, to bring smallpox to the forefront of national health agenda at the outset of the 1960s. These impulses would go on to get support from an unexpected source: a military government that came to power after a coup in 1964, which was keen to get international recognition and a national smallpox eradication campaign provided such an opportunity.^22^ Sanjoy Bhattacharya’s case studies of independent India assess the complexity of its national and local governments’ engagements with WHO officials seeking to promote the interests of the worldwide SEP in the 1960s and 1970s. Highlighting complex fragmentations and alliances within governmental and WHO structures, he presents us with descriptions of intricate networks of international, national and district-level public health workers with varying loyalties and, therefore, strategic approaches.^23^ Bhattacharya reveals how overseas workers and, by implication, the political interests they represented struggled to dictate any terms to Indian central and state governments, which kept a firm grip on pilot investigations as well as subsequent mass vaccination and search and immunisation practices (including the work carried out in the kingdom of Bhutan).^24^ Vivek Neelakantan provides a carefully researched assessment of the challenges of ridding Indonesia, a multi-island nation, of smallpox. In this, he presents us with detailed descriptions of programmatic specificities across complex political and social structures, as well as the national and international collaborations that underpinned successes.^25^ However, this work does not contain a detailed assessment of the intricate international exchanges and deals underpinning the design, expansion and workings of the first stage of the SEP between 1958 and 1967, which is provided in the following sections.

## 2 Founding the Worldwide Smallpox Eradication Programme

It is important to remember that the first WHA of 1948, held in Geneva, identified smallpox as one of the public health priorities requiring attention from the new international collective brought together by WHO frameworks. To this purpose, WHA Resolution 1.16 was prepared and opened up for a vote; a majority mandate resulted, indicating that there was general support for organised action against this disease, which was a major concern especially within decolonising countries in what would soon become the WHO’s South East Asia Region (the regional office, WHO SEARO, was formed the same year). This WHA decision caused the WHO Expert Committee on International Epidemiology and Quarantine to form a dedicated study group to assess the impact and control of smallpox, and this body worked to ensure that these issues remained visible within WHO structures worldwide. As news about the large-scale production of new, effective and freeze-dried smallpox vaccines circulated within health agencies, the disease stood out among other communicable diseases as a potentially solvable problem. In 1950, the committee overseeing the PAHO (the WHO regional office for the Americas), voted to start regional smallpox eradication efforts.^26^ In the same year, the third WHA recommended, through Resolution 3.18, which was passed through with the help of another majority vote, that more weight should be given to smallpox control in the WHO’s regular programme and budget. In 1953, Dr Brock Chisholm, then WHO DG, proposed a worldwide smallpox eradication campaign and this was considered within the sixth WHA, where WHO member states combined to recommend that WHO regional offices, national governments and members of WHO expert advisory groups study the issue collaboratively.^27^ Chisholm presented the results of the consultations to the thirteenth meeting of the WHO HQ’s Executive Board (EB) in early 1954. The results were discouraging to those seeking more ambitious and organised action against smallpox. Reporting on the meeting and the seventh WHA that followed it, Dr Melville Douglas Mackenzie, a British WHO EB member, noted that ‘opinion was by no means unanimous for a campaign at the present time, as would be noted from the views expressed by the various regional committees [the bodies that the new WHO regional offices reported to]’.^28^ Even though the French delegate had support when he noted that there had been progress in reducing smallpox incidence through organised mass vaccination campaigns, representatives from other countries doubted that eradication was technically feasible; they asked why some countries continued to have endemic smallpox despite the existence of major vaccination drives.^29^ Significantly, however, these disagreements did not result in the abandonment of plans for a campaign aiming to rid the world of the disease. There was still sufficient synergy within the WHA to argue for more research into future smallpox eradication strategies, and national governments were urged to keep up mass immunisation work as part of their general public health programmes.^30^

Four years later, at the eleventh WHA, held in the US city of Minneapolis in May 1958, the Soviet Union’s delegation, led by Dr Victor Zhdanov, tabled a resolution urging smallpox eradication.^31^ This called for a five-year worldwide SEP, initially directed at countries where the disease was endemic. To rally support, the USSR offered to donate twenty-five million doses of freeze-dried vaccines.^32^ This proposal received a majority vote, which Nancy Stepan attributes to a general keenness to welcome the USSR back into formal WHO membership (the country and several of its Warsaw Pact allies had left this UN agency in 1948).^33^ A careful assessment of published and unpublished WHO papers, such as those relating to behind the scenes negotiations between officials, reveals a significantly more complex picture. The Soviet representatives made their case by describing how the USSR had eliminated smallpox throughout the country in the 1930s with the help of compulsory vaccination and the use of heat-stable, freeze-dried vaccine, which had allowed for effective immunisation even in warm and subtropical climatic conditions. At the same time, Soviet negotiators were brutally honest about their subsequent failures, pointing out that they had been unable to stop importations from neighbouring countries with endemic smallpox, despite great investment into structure for disease surveillance, containment and prevention. Therefore, they presented worldwide smallpox eradication as the only way for governments to reduce the great outlays of money and people needed to protect their populations. The Soviet resolution for such a comprehensive SEP then proposed the running of mass immunisation campaigns with effective vaccines in all smallpox endemic countries for five years, even though it admitted that eradication was likely to take at least a decade. This report from the USSR also suggested that the WHO share costs of purchasing vaccine and building the necessary teams with national governments.^34^

When one researches the Soviet proposal’s provenance, and looks at the negotiations that attended the creation of different drafts, it is obvious that it was unveiled at the WHA after considerable diplomatic preparation and exchange, usually carried out behind closed doors; these complex processes have not been recognised, researched and contextualised in the existing scholarship dealing with the SEP. The USSR government shared the first version with supporters within the WHO HQ’s bureaucracy on 6 March 1958, recommending to the WHO DG that he create a special section within the WHO HQ’s Department of Advisory Services to support member states with smallpox control. Interestingly, while there was general support for the upcoming WHA resolution within the WHO HQ, the Soviet recommendation was taken out from the final version tabled on 6 June 1958.^35^ The USSR’s proposals received detailed consideration during the fifteenth and sixteenth sessions of the influential WHO Committee on Programme and Budget, held on 11–12 June 1958, and involved senior WHO HQ and regional office representatives. The suggested plans for action were unanimously accepted. Delegates from Ecuador and the Netherlands even declared that smallpox eradication was as important as malaria eradication, musing that the newly advocated programme was perhaps more feasible. Sudan’s representative argued that the WHO had an important and unique international role to play, in that its structures around the world could deliver a co-ordinating role that was otherwise impossible to deliver, with its officials operating across borders of neighbouring countries. There had been major smallpox outbreaks around the world in 1957 and 1958, and memories of the horrors of the disease and the pressures they placed on public health services were fresh in the delegates’ minds; these were flagged up by Ecuador’s and Vietnam’s representatives. By contrast, the Australian and Swiss delegates were more cautious, and they underlined the scale of resources that would be required for such an expansive programme. They argued that formal WHA clearances for the SEP needed to be denied until all endemic countries had committed the necessary resources. Ireland’s and New Zealand’s representatives supported such caution, and submitted amendments to the draft Soviet resolution asking the WHO DG to collect information on the financial, administrative and technical implications of the proposed national smallpox eradication campaigns that would underpin a worldwide programme.^36^

The WHO DG responded by preparing a report for the next WHO EB meeting in January 1959 and the WHA that year. This was based on replies to questionnaires sent out by his office to all member states to assess diversities in smallpox epidemiology, appetite for more expansive vaccination measures and the level of resources they were willing to commit. Representatives of fifty member states replied, but not all of these were from smallpox endemic countries. This forced the WHO DG to declare that some of his findings were incomplete, especially in relation to available financial resources. Nevertheless, he used this report to propose that all future national smallpox eradication campaigns should consist of structures for mass vaccination, health education and disease surveillance. He also announced that each country would be responsible for its campaigns, in line with national legal regulations, and they would need integration into general public health services. The WHO offices in Geneva and the regions would, when requested, provide expert technical advice and help co-ordinate campaigns across international borders.^37^ Dr P.M. Kaul, the WHO Assistant Director-General from India, was tasked with introducing this report to the WHO EB and WHA. He accepted that there were considerable financial and organisational problems that needed to be overcome. However, he also highlighted the positives of creating political synergies, declaring that:

There could be no doubt that such a determined effort was worthwhile and opportune because, if the campaign were successful, heavy annual expenditure by individual countries would become unnecessary. With adequate support and co-operation from national health authorities and international assistance, considerable progress towards eradication could be achieved in a relatively short time.^38^

The WHO EB meeting of June 1958 created a special account for smallpox eradication within the WHO HQ, whose managers solicited donations of money and vaccine stocks from around the world.^39^ When one looks at the negotiations about SEP budgets, it is important to recognise that initial financial outlays were primarily mobilised nationally and regionally.^40^ For example, the African Conference on Smallpox Eradication hosted by the WHO regional office for Africa in Brazzaville, Congo, highlighted the importance of coordinated smallpox eradication services, which involved recruiting and training of personnel, vaccination techniques and health education campaigns for target populations. There was agreement that cross-border work was necessary, since populations and the causative virus did not respect political maps. Also acknowledged was that African territories and countries would require material aid from international agencies in order to prepare vaccination structures for the needs of eradication.^41^ Similar perspectives emanated from other regional contexts as well. Over the course of 1958 and 1959, the WHO regional office for the Eastern Mediterranean (WHO EMRO) deployed a team composed of an epidemiologist and a laboratory expert to carry out surveys in all its member states, which reported a range of practical challenges as it advocated the strengthening of smallpox vaccination services.^42^ This was akin to activities within South Asia, reported and carefully considered for its wider relevance at the WHO Inter-Regional Smallpox Conference hosted by WHO SEARO in New Delhi in November 1960.^43^

All these investigations underlined the need for good quality, thermostable freeze-dried vaccine, which, in turn, raised serious questions about and for smallpox endemic countries, where supplies were incomplete and unreliable.^44^ The WHO HQ started setting aside fellowships in response, as well as funds for training courses and the secondment of consultants, for projects that would strengthen supplies of reliable vaccines and other SEP goals. A handful of laboratories in developed countries, such as the UK’s Lister Institute of Preventive Medicine, were identified as sources of such technical assistance and training facilities.^45^ The process of distributing fellowships was not straightforward. Correspondence between Dr Douglas McClean of the Lister Institute and Dr A.M. Payne of the WHO HQ’s Section of Endemo-epidemic Diseases in August 1958 highlighted the prolonged and complicated nature of discussions about the need to identify one or two regional centres responsible for vaccine production and training. The Lister Institute wished to accommodate no more than one or two fellows from each WHO region, after difficulties with two applicants from Iraq and Sudan nominated by WHO EMRO. The Institute, therefore, wished to disallow candidates from ‘countries that lacked the necessary scientific resources and which would be likely to bring the method of vaccine production into disrepute’.^46^ Some months later, a placement request from WHO HQ for a South Korean veterinary officer responsible for national vaccine production resulted in further tensions. Professor A.A. Miles, the director of the Lister Institute, questioned the suitability of receiving candidates from a ‘small country’ and WHO’s inability to send groups of two or three fellows at a time for training.^47^ In an effort to change Professor Miles’s mind, the WHO HQ requested external experts to prepare assessments of the candidate’s curriculum vitae and Seoul’s vaccine production facilities. As their positive feedback was relayed back to the Lister Institute, it was also underlined that South Korea was one of the few countries in the region still affected by smallpox, and that the production of high-quality vaccine would help both the country and the region to fight this disease.^48^ Following further negotiations, the South Korean candidate was offered the opportunity to hold a WHO fellowship at the Lister Institute from February 1959 onwards.^49^

In many ways, the WHO HQ negotiators’ stubbornness and the Lister Institute’s ultimate acquiescence underlines how the WHA’s resolutions supporting smallpox eradication had created a legally binding agreement that was being deployed internationally to some effect. These legal devices were used by coalitions of WHO and government officials to push for the restructuring of national vaccination and disease surveillance frameworks, even if the impact across and within countries was uneven. Some national governments responded by launching pilot eradication projects, which, their representatives argued, were needed to establish the most effective methodologies and prepare reliable financial estimates of the cost of a wider SEP. Many commentators regarded South Asian countries, whose governments worked closely with the WHO SEARO and WHO EMRO, as the most important sites for such investigative drives as they reported the highest incidence of smallpox.^50^ The Indian Council of Medical Research set up a committee in May 1958 to examine the possibility of smallpox eradication, and the central government accepted its proposal that investigative pilot projects be set up across the country.^51^ Between 1960 and 1961, such projects were run in one district of each Indian state, which provided government officials at all levels of national administration with first-hand experience of many practical challenges. This work also helped the collection of the evidence base that justified by the launch of the National SEP in January 1962.^52^ Similar activities were organised in East Pakistan (Bangladesh), another densely populated endemic region. Pilot projects were launched in the two districts most affected by smallpox during the epidemic of 1957 and 1958, that is, Commilla and Faridpur. With 4.4 and 3.2 million inhabitants respectively, the task was difficult and blanket immunisation required eight months of concerted work. Regarded a success upon completion, this campaign also provided Pakistan with the evidence base with which to justify and formally launch its own national smallpox eradication campaign in November 1961.^53^

As these SEP pilots developed, they helped uncover disparate, ground-level problems to the programme’s international advocates, and successive WHA meetings considered the best means of countering these challenges. The thirteenth WHA, held in 1960, saw the Soviet representative question the accuracy of the epidemiological data presented in the WHO DG’s report and suggest the immediate development of special surveys that would allow the collection of more reliable information.^54^ At the fourteenth WHA, the Peruvian delegate highlighted financial difficulties and suggested a draft resolution that would make the WHO DG allocate money for the SEP through the WHO HQ’s regular budget. Dr W.A. Karunaratne from Ceylon (Sri Lanka), as chair of the meeting, noted that the WHA had just committed funds for malaria eradication for the next three years and highlighted concerns that support for the SEP would require additional national contributions to WHO budgets. This led to a withdrawal of the Peruvian draft resolution, which kept WHO smallpox eradication budgets at previous levels.^55^ At the fifteenth WHA, Thailand’s delegate wondered how more reliable vaccines could be developed and made generally available.^56^ The WHO DG reported that five countries had donated thirty-four million vaccines and that all but 6.5 million had been used.^57^ The delegate from Upper Volta (Burkina Faso) queried the claim, complaining that his government did not receive stocks during an epidemic outbreak in 1961 and therefore had to import vaccines from France at great expense.^58^ As this meeting progressed, it became clear that the lack of good quality vaccine was only one problematic factor. The WHO DG highlighted the lack of investment in SEP-related administrative structures, underlining the need for greater international assistance for developing country contexts.^59^ The Indian representative argued that the problem was not lack of technical expertise or vaccines, but an insufficiency of vehicles and refrigeration equipment. Delegates from Sudan and Pakistan made similar arguments.^60^ At the sixteenth WHA, the delegate from the United States proposed that the WHO DG make provision of US$10 million for SEP through the regular budget. Despite receiving support from several delegates from African nations, the proposal failed to pass with a majority vote.^61^

Countries free of smallpox, especially those in Europe, articulated other concerns. Reference was made to the large sums of money and volumes of staff time being used for surveillance and quarantine measures, which were considered necessary in a situation where air travel across continents became faster and more accessible (by 1962, there were twenty-nine million people travelling by aeroplanes).^62^ Several smallpox importations, which caused wider outbreaks, were linked to air travellers arriving in the United Kingdom, West Germany, Sweden, Poland and Canada in the 1960s. The Dutch delegate to the 1963 WHA suggested that the WHO HQ should utilise the anxiety about future outbreaks to rally wide-ranging support for the SEP.^63^ In December 1964, the Norwegian authorities were rattled by an outbreak in Sweden and started planning wide-ranging preventive action. The Director General of Health Services of the Royal Norwegian Ministry of Social Affairs contacted Dr Chandra Mani, WHO SEARO’s director, requesting his help to get permission from the Indian government for a three week visit by a Norwegian academic, who would study clinical aspects of smallpox, in January 1965.^64^

As multiple anxieties about the international spread of smallpox coalesced in the first half of the 1960s, interest groups within countries came together within bodies like the WHA to develop a wider SEP. This led to further collaborative research, whose outcomes would go on to shape future programmes. Between 1960 and 1965, the WHO HQ spent around US$65 000 supporting different aspects of smallpox research, which covered topics as diverse as vaccine production, disease epidemiology, applicability of hyper-immune gamma globulin as a basis for curative treatments, and the variability of the impact of different variola virus strains. These research activities were multi-sited, developed and deployed across countries and WHO regions, involving institutions that ranged from the Lister Institute of Preventive Medicine in the United Kingdom to hospitals across Madras (Tamil Nadu) state in India and multiple countries in western Africa.^65^ To assist in the assessment and communication of findings, as well as the translation of research into new policy initiatives, the WHO HQ formed a committee of international experts. This body met in January 1964, discussed the findings of the research commissioned by WHO offices in partnership with governments, and provided a blueprint for the intensification of action.^66^ Their report asked countries to strengthen their structures for mass immunisation and change vaccination methods, even as it highlighted the problems caused by the use of ineffective vaccines. The committee’s recommendations received wide-ranging support and in February 1964, one month after its meeting, Dr A. Saenz from the WHO HQ’s Virus Unit attended a meeting at the Swiss Paediatric Society to discuss the viability of using sub-cutaneous and intra-cutaneous vaccination.^67^ Following this, the WHA passed Resolution 17.431964 in 1964, which requested the WHO DG to prepare a further, comprehensive plan for the worldwide smallpox eradication based on the evidence presented by the Expert Committee.^68^

The WHO HQ’s response was to announce that it would work with member states to prepare detailed surveys of conditions in endemic regions; the aim was to develop a fulsome understanding of challenges faced by national administrations and create workable solutions. Visits to Afghanistan, Burma, Mali and Nigeria were organised in late 1964 and early 1965. The shortage of national funds and public health personnel was presented as the biggest stumbling block, and investigators in Mali noted that smallpox was just not regarded a priority and was considered less important locally than the ongoing, USAID-supported measles control programme.^69^ Similarly, WHO assessors in Afghanistan noted the lack of national political support for smallpox eradication and raised serious doubts about local medical officers’ commitment to the programme. Interestingly, officials in WHO HQ decided to edit out such criticism from draft reports in order not to jeopardise future efforts at mobilising administrative support for SEP.^70^ All these surveys highlighted the need to rally wide-ranging international and national support for the goal of smallpox eradication. In 1965, smallpox was made the theme of the World Health Day celebration across WHO frameworks, and President Lyndon Johnson of the USA publicly expressed his support for SEP. This allowed the USAID and the US CDC, which had been working on mass measles immunisation in western Africa since the early 1960s, to develop a joint measles and smallpox eradication programme for twenty African countries. Dr Marcolino Candau, who had been elected WHO’s DG in 1953, negotiated US government buy-in into these plans, flying to Washington DC to assure US partners about the organisation’s support.^71^ Resolution 18.38 followed at the eighteenth WHA in 1965, which asked the DG to mobilise further bilateral assistance programmes for an expanded, worldwide SEP.^72^ A smallpox eradication unit was formed within the WHO HQ to co-ordinate international activities, and Donald A. Henderson and Isao Arita assisted Candau in preparing a detailed plan. This argued that SEP would need a budget of US$180 million and that thirty per cent of this sum would come from international funds. Candau proposed that at least forty per cent of this would, in turn, come from the WHO’s regular budget, which would represent a sharing of expenses by all member states.^73^ Discussions at the thirty-seventh WHO EB meeting in January 1966 and the nineteenth WHA that May reveal that while there was unanimity regarding SEP expansion, disagreements remained about financing. The British delegate to the assembly proposed a US$1 million limit in spending and suggested programme postponement if this were not possible. Despite this, support from a collective of African, South American, Scandinavian and eastern European countries meant that there was a majority WHA vote for an escalation of SEP work.^74^

## 3 South America and the Justification of a Worldwide SEP

Existing institutional, WHO-related histories of the birth, expansion and completion of the SEP in the Americas highlight the wider influences of an outbreak in New York, USA, in 1947.^75^ Linked to an importation of the disease from Mexico, the episode led to eight deaths and the vaccination of 6.3 million people in one month.^76^ In response, Dr Fred Soper, then WHO PAHO’s director and a strong supporter of the idea of disease eradication, became interested in thermostable freeze-dried smallpox vaccines produced in Europe.^77^ According to the WHO HQ’s official history of smallpox eradication of 1988, this vaccine was considered efficacious for tropical regions across the Americas, which were seen as sources of variola reservoirs and a public health threat to smallpox-free countries like the USA.^78^ In 1949, Soper proposed a smallpox eradication programme for the two American continents, receiving the support of WHO PAHO’s Executive Committee.^79^ The thirteenth Pan American Sanitary Conference in 1950 endorsed the proposal and made a relatively modest US$75 000 available for work in 1952.^80^ Unpublished papers about this plan and its national iterations shows this funding being used mainly for the provision of technical advice and equipment for freeze-dried vaccine production by member states.^81^ However, academic scholarship has generally not examined the impact of the WHA resolutions in support of SEP from 1958 and 1959 on the reshaping of international, regional and national work on variola surveillance and vaccination in South America. These WHA-level developments led to new negotiations between the WHO HQ, WHO PAHO and member states, as well as new forms of preventive work and evaluation that involved WHO representation within several countries. These activities were carefully studied across WHO frameworks, with these analyses feeding into their engagements with the WHA. As the evidence provided here shows, the results of this work fed into the explanatory frameworks that justified the so-called intensification of the SEP over the course of 1967 and 1968.

The great complexity of SEP negotiations within and across specific South American country contexts is best assessed through unpublished documentation, particularly comprehensive files that include field reports and related assessments. These, more than diplomatically oriented published reports or unpublished briefing documents for large international gatherings, provide detail about disagreements, concerns about problems, ambitions for reform and complex negotiations on all these issues. All these engagements were also interconnected with the preparation of evaluations that many WHO officials hoped would help justify an expansion of international action. Studied carefully, such historical materials help us properly assess the great variety within – and the intricate connections between – national and international administrative structures. For example, detailed paperwork prepared by WHO PAHO and WHO HQ officials working with certain South American member states after the WHA’s 1958 SEP resolution reveals qualitative shifts in negotiations, preparation of new proposals for presentation to different governments and an awareness that national disparities would ensure variations in reception, activity and impact within and across countries.^82^

Such shifting arrangements were clearly visible within several South American governmental contexts. For example, Peru responded to WHO PAHO’s 1950 call for smallpox eradication almost immediately, starting its programme the same year with some success in limiting the prevalence of the disease within its territories.^83^ Based around plans to vaccinate eighty per cent of the population in five years, the programme was run up to 1953, with freeze-dried vaccine produced by Michigan State Laboratory in the USA (sourced and paid for by WHO PAHO), as well as locally produced vaccines produced with equipment donated by the regional office. Evaluations revealed the country free of endemic smallpox in 1955 and the work that made this possible was proudly advertised at a major international meeting held the following year in Lima.^84^ The Peruvian government remained involved in post-SEP launch discussions in 1958, drawing on its expertise. However, following smallpox importations from Brazil in 1963, the country actively joined regional SEP efforts.^85^ Investigations linked the outbreaks to weak rural vaccination services, which had allowed the development of pockets of unimmunised populations; the response to the outbreaks resulting from these cases took the shape of new mass immunisation campaigns, which were launched successfully between 1964 and 1966.^86^ Venezuela witnessed similar trends. Mass vaccination campaigns encouraged by WHO PAHO’s 1950 call helped rid the country of smallpox in 1956.^87^ Although this initially caused a half-hearted engagement with the SEP of 1958 and 1959, importations from the Brazilian region of Gran Sabana in June 1962 ensured a more fulsome engagement with the new WHA-advocated smallpox eradication work; the resulting deployment of comprehensive vaccination, search and containment campaigns helped bring the situation under control.^88^

Different trends existed elsewhere in South America. Bolivia started its mass smallpox vaccination campaigns in 1958, after an epidemic in the previous year made its government more amenable to the WHA’s call for SEP.^89^ This helped mobilise the *Servicio Cooperativo Interamericano de Salud Publica*, an international agency jointly created by the Bolivian, North American governments and USAID, and linked to WHO PAHO.^90^ However, as rural vaccination remained incomplete and inconstant, importations linked to Brazil kept stoking new outbreaks,^91^ and this led to a new agreement with WHO PAHO in 1963 that created a new and comprehensive mass immunisation programme.^92^ Similarly, Paraguay was assisted by WHO PAHO to run mass vaccination campaigns between 1958 and 1960, leading to the country becoming free of smallpox between 1961 and 1963.^93^ However, the country reported seven importations from the Brazilian state of Mato Grosso in 1964 and another such case in 1965.^94^ A mass vaccination campaign covering around sixty per cent of the population followed and this enabled the country to avoid further infections.^95^ Similar trends were visible in Colombia and, by 1966, WHO PAHO was using data from this country to emphasise the importance of strong basic health services to smallpox vaccination after the so-called attack-phase.^96^ Argentina, South America’s second largest country, began its organised fight to eradicate smallpox in 1960, in response to an outbreak of sixty-five cases.^97^ Targeted vaccination between 1960 and 1963 covered about thirty per cent of the national population, while immunisation activities in 1964 and 1965 were concentrated inside provinces bordering Brazil and Paraguay (the latter drive led to 5 530 000 vaccinations in the provinces of Corrientes, Misiones, Chaco, Formosa and Salta).^98^

As an identified source of smallpox importations across other South American countries, Brazil received significant attention from WHO PAHO, WHO HQ and the WHA. In 1956, President Juscelino Kubitschek argued that smallpox was under control in his country, and declared that diseases like malaria, yaws, worm infestations and tuberculosis were the government’s greatest priorities.^99^ A gradual shift in Brazilian government attitudes started in 1958, after countries like Ecuador, Bolivia and Paraguay supported the WHA’s call for SEP. That year, Kubitschek spoke to the Brazilian National Congress, declaring that smallpox eradication was both necessary and possible.^100^ Following this, the country signed up to Resolution VI of the fifth Pan American Sanitary Conference in 1958, which declared that the eradication of smallpox was a public health necessity for the Americas.^101^ In the same year, WHO PAHO’s representative in Brazil visited the Institute Oswaldo Cruz in Rio de Janeiro, and offered financial and technical advisory support necessary for starting the production of freeze-dried vaccine. In 1959, Dr Jose da Cunha, responsible for vaccine production within the Institute, was awarded a WHO PAHO fellowship that enabled him to visit major manufacturing facilities in the USA, the United Kingdom, Germany and the Netherlands.^102^ By 1961, the Brazilian government had established a new National Health Code, which declared that the government would convert disease control projects into wider eradication programmes as soon as conditions were favourable, and specifically mentioned the importance of ridding the country of smallpox.^103^ New federally financed but locally managed immunisation initiatives followed, which led to the vaccination of 2 600 000 people in eighteen of twenty-six Brazilian states.^104^ In January 1962, Brazil’s federal authorities created the National Campaign Against Smallpox (NCAS), providing its component team with freeze-dried vaccine made in Rio de Janeiro, Porto Alegre and Pernambuco.^105^ The stated goal was to vaccinate eighty per cent of the population in five years, involved some central government officials, but continued to place greatest responsibility for delivery and evaluation on state governments.^106^ The results of this programme were discussed at the third National Health Conference in 1963, where the importance of smallpox eradication was highlighted.^107^ Attendees reported financial challenges at this meeting, which were connected to political instabilities that would go on to contribute to the military coup of 1964; the federal Ministry of Health underwent almost constant restructuring, which, in turn, undermined political support for smallpox eradication in 1963.^108^

As the new Brazilian military government sought international legitimacy, a bigger and more visible smallpox programme provided new opportunities for such diplomatic engagement. Brazil, WHO HQ and WHO PAHO came together around a new agreement in October 1964, which promised provision of further technical and advisory assistance from Washington DC and Geneva for national eradication initiatives.^109^ Plans for a new Brazilian Smallpox Eradication Campaign (SEC) emerged from these developments, as well as a review of NCAS data, which showed that only three states – Sergipe, Rio Grande do Norte and Pernambuco – had vaccinated eighty per cent of their population.^110^ NCAS’s decentralised structures were dispensed with by the new government, which favoured increased control from within the national capital.^111^ The Brazilian federal health minister acknowledged in 1966 that the country was ‘regrettably among the most important smallpox foci in the world and the most relevant in the American continent’.^112^ On 22 August 1966, just days before the SEC was to be launched formally, Dr Hector Acuna, representing WHO PAHO in the country, wrote to Dr Karel Raska, then chief of the Communicable Disease Division at WHO HQ, highlighting worries about the lack of administrative and financial capacity within Brazilian states to carry out mass vaccinations.^113^ Raska responded by suggesting that separate plans for central and state governments be prepared, and suggested that precedents from Pakistan be used as supporting evidence for such an action.^114^ Acuna’s response was firmly negative and he underscored the impossibility for proposing separate plans for the states to the country’s central government, even as he warned against the diplomatic and strategic dangers of equating the country with Pakistan.^115^ This ensured that WHO PAHO ended up with a relatively marginal role, and only three of its medical officers and one statistician received permission to join the SEC in 1967.^116^

Such administrative dissonances ensured that smallpox outbreaks continued across South America, as porous borders allowed infections to spread from one country to another. It is, of course, important not to over-estimate WHO PAHO’s power to contribute to national SEPs across the Americas. When the regional office decided to push SEP priorities more vigorously in 1964, internal WHO correspondence revealed that it did not have the necessary resources to make lasting contributions to member states in the continent. The regional committee, therefore, passed a resolution that year authorising WHO PAHO’s director to request and accept contributions of money, equipment and personnel from donors around the world, for redistribution from Washington DC to different national governments.^117^ Sufficient resources were mobilised to allow the regional office to embark on a survey on the smallpox situation in January 1965, which took three months to complete. The report concluded that the disease remained endemic in Brazil, Argentina, Colombia, Paraguay and Peru, attributing the situation to insufficient finances, low vaccine quality and supply, and the lack of uniformity and continuity in vaccination programmes. Weaknesses in basic health services and related surveillance systems were blamed for the reintroduction of smallpox into Paraguay and Peru, after their officials had stopped endemic transmission. The continuing challenges in limiting the spread of the disease in the Amazon area, where tribal populations moved freely across national borders, was underlined. Significantly, this landmark report, which would go on to form the regional evidential bulwark for the intensification of smallpox eradication, used data from the period between 1950 and 1966 to argue that mass vaccination had worked to drive down disease incidence.^118^

Despite all these efforts at consolidation, South America continued to report smallpox. Concerns caused by the confirmation of twenty-one cases in Argentina in 1966 led to the signing of a new agreement between its government and WHO PAHO in March 1967, which committed the country to the so-called intensified phase of worldwide smallpox eradication.^119^ Strikingly, the country joined on its own terms. Although the WHO HQ and WHO PAHO initially asked that only freeze-dried vaccine be used, the Argentine government negotiated the deployment of nationally produced, therefore cheaper, glycerinated vaccine.^120^ Colombia was considered to be in danger from importations, causing its government and WHO PAHO to sign an agreement to intensify SEP efforts in May 1967.^121^ Peru’s authorities agreed to a similar tie-up that August.^122^ WHO PAHO negotiated similar agreements with Paraguay and Ecuador the same month.^123^ As new initiatives launched from 1966 onwards helped to bring about falls in smallpox incidence in South America, these datasets empowered groups of WHO officials to make a stronger case for an intensified SEP.^124^ This comes through powerfully in the recommendations made in 1968 by the WHO scientific group on smallpox eradication, which justified intensification by using epidemiological and programmatic data from 1958 onwards (see Table 1).^125^

**Table 1:** Annual number of smallpox cases by continent, 1959–66.^**^

Continent	1959	1960	1961	1962	1963	1964	1965	1966	1967^*^
Africa	16 307	16 823	26 060	24 329	16 863	12 506	16 784	14 127	9 554
Asia	71 309	39 843	53 957	63 616	98 784	43 537	39 145	50 494	50 958
Europe	26	47	24	136	129	–	1	71	3
North America	–	–	–	–	–	–	–	–	–
South America	5 490	7 931	9 026	9 718	7 151	3 398	3 515	3 092	426
Oceania	–	1	–	–	–	–	–	–	–
Total →	93 132	64 645	89 067	97 800	122 927	54 441	59 445	67 784	60 1941

* Until 15 July 1967.

** Consolidated data compiled by WHO from various sources

Source: *Smallpox Eradication – Report of a WHO Scientific Group: World Health Organization Technical Report Series, No. 393* (Geneva: WHO, 1968), 7, Official Publications Room, University of Cambridge Library, Cambridge, UK.

## 4 Conclusion

As with all major WHO-supported programmes, the SEP operated at different levels. While the secretariat of the WHO HQ reported to the WHA and drew up action plans in consultation with its EB, it could never avoid discussions with governments of member states about the acceptance of proposals and day-to-day work. In these engagements with countries, the WHO HQ’s secretariat had to rely on WHO regional offices, which generally managed negotiations about adaptations of general policy to specific national legal frameworks and infrastructural conditions. While representatives of the WHO HQ could request consultations with senior government officials, the daily intricacies of health governance was almost always left in the hands of WHO technical staff working through the regional and country offices and their counterparts inside national administration. The WHO DG could propose ideas and provide suggestions to a regional director, but the latter also remained answerable to a regional committee and its constituent member states. In terms of the SEP, this ensured that top-down imposition of policies remained impossible. Continual negotiations were necessary at all levels of WHO administration before any administrative shifts could take place, and the situation was complicated by the fact that these were connected to similar engagements with complex structures of national governance. Recognising such features in international health administration helps in the adoption of an approach that de-centres the history of worldwide smallpox eradication, ensuring it is necessarily wider than focused narratives about the US government and actors in western Africa and further afield from the mid-1960s onwards. An approach that accommodates the study of multiple actors and their engagements allows the recovery of a richer history of the SEP, which can be connected to processes of decolonisation post-1945 and events surrounding the WHA of 1958.

Recognition of these complexities in the design and implementation of international health has helped us collect, analyse and present a range of previously unused WHO papers. This material shows us that several pilot programmes around the world and their many avatars produced epidemiological and administrative data that helped buttress arguments for increased support for the SEP. Indeed, it could be argued that such datasets, collected between 1958 and 1967, proved crucial to the primary argument that variola could be eradicated. A move away from narrow institutional histories, and a focus on unpublished files dealing with engagements between the WHA, the WHO HQ, WHO regional offices and its member states, provides a number of fresh perspectives. Despite programmatic variations, highs and lows within and across regional and national contexts, this body of information shows that focused mass vaccination programmes did help engineer major reductions in smallpox incidence in several endemic countries. For example, Burma (Myanmar) implemented the SEP successfully between 1958 and 1966, despite political and economic difficulties, with the help of good rural basic health services and assistance from the WHO regional office for South East Asia and WHO HQ; the country was confirmed as being free of smallpox in 1966.^126^ Similarly, several political formations within the WHO Western Pacific region (like the Federation of Malaysia and the two sections of Vietnam) and in the WHO EMRO (such as Saudi Arabia, Iraq and Iran) were reported as being largely smallpox-free by 1967, after successful implementation of their own distinctive SEP campaigns.^127^ Reports noted dramatic reductions in smallpox incidence in Africa in 1966 and 1967, including contexts like the Ivory Coast and the Central African Republic, where local staff had worked to deliver plans co-operatively developed with international actors like the WHO HQ, WHO regional office for Africa, USIAD and CDC to interrupt endemic smallpox transmission.^128^

Such assessments created expansive networks of solidarity between groupings of WHO and government officials around the globe, who then used this data to signpost strategic failures, future possibilities and the usefulness of the wider adoption of results from successful experimentation with vaccination strategies carried out worldwide.^129^ Such datasets continually enabled justification of the creation of new SEP structures across WHO regions.^130^ For example, in response to arguments about administrative weaknesses, the WHA and WHO HQ released funds in 1963 that allowed the mirroring of the Smallpox Eradication Unit in WHO HQ within WHO regional offices.^131^ Similarly, when WHO and government officials working across South Asia insisted in 1964, on the basis of evidence collected since 1958, that it was important to ensure that work in countries and across their borders was synchronised (such views had also been advocated from within the Americas), necessary legal agreements were negotiated and put into place.^132^ Evidence drawn from across WHO regions between 1958 and 1966 was also used to make the argument that the SEP’s future success would depend on an improvement of smallpox surveillance and reporting structures,^133^ which was, in turn, linked to concerted efforts to promote the strengthening of basic health services.^134^ Negotiations that followed with national governments did not result in the uniform or lasting acceptance of all ideas and practices, but references to concrete examples of success since 1958 allowed SEP advocates to keep its different national chapters on track, as a WHA-backed initiative, throughout the 1960s.^135^

Importantly, this contributed to a situation in 1967 where WHO officials – employed through its regular budget, seconded to the organisation by governments from around the world or funded by special programme grants – were able to fan out globally to work with different national chapters of the SEP. As they embarked on their new brief, these workers were operating in diplomatic and public health spaces created by the projects run between 1958 and 1966 to carry out further investigations, report problems, discuss possible solutions and develop policy adaptations in response to a multiplicity of political, social, economic and cultural conditions. Such diverse engagements always required negotiation at different levels of governance, which was, once again, made possible by administrative structures created as a direct result of the international action on SEP between 1958 and 1966.^136^ The importance of the data collected between 1958 and 1966 is also demonstrated through the deliberations within the Indian Directorate General of Health Services, which organised a meeting in New Delhi on 22–29 of January 1968 that involved representatives from all states and union territories.^137^ Based on its recommendations, the Government of India decided to renew its commitment to the SEP, and collaborate with the WHO HQ and regional office, even if senior WHO negotiators continued to worry about the resilience of these promises.^138^

To conclude, the use of a more expansive timeline for the SEP allows us to recover details of complex projects carried out in a variety of geographical contexts that have generally not received scholarly attention. This, in turn, allows the preparation of more inclusive and independent histories that do not end up promoting narrow institutional interests. This approach enables the study of the SEP’s past in its own terms, as a complex programme that involved hundreds of thousands of national and international workers who struggled together for over two decades to deliver a goal that many had considered impossible. If history is to ethically inform current and future policy, the methodology used here can assist the development of more fulsome appreciations of intricate administrative and political challenges that await international coalitions as they plan campaigns that have to negotiate wide-ranging disparities that exist on the ground. Critical historical analysis, at the very minimum, informs those charged with public health leadership that their work cannot be based on an inflexible, top-down enforcement of narrow sets of ideas and practices. Rather, detailed historical research points to the centrality of the need to respect variations in attitudes, as well as a willingness to negotiate with wide-ranging actors on equal terms. This, in turn, promotes a move away from damaging and artificial civilisational narratives that often mark institutional histories, where one group of people is presented as educators who uplift everyone else from a state of scientific ignorance to one of supposed technical certainty and unanimity.^139^ Inclusive histories of international and national governance, like this article, do not consciously privilege some voices over others, and can help stoke respectful dialogue with the many actors responsible for national public health policy design and implementation.

## Footnotes

1.
World Health Organization, *The Global Eradication of Smallpox: Final Report of the Global Commission for the Certification of Smallpox Eradication, Geneva, December 1979* (Geneva: World Health Organization, 1980), 56–60.

2.
World Health Organization, *Thirty-Third World Health Assembly: Verbatim Records of Plenary Meetings Reports of Committees* (Geneva: WHO, 1980), 157.

3.

Horace G. Ogden , CDC and The Smallpox Crusade (Washington DC: US Department of Health and Human Services, Centers for Disease Control, 1987).

4.
The lack of attention paid to diversity, in terms of gender and race, in these historical explications is quite staggering, even when roles played by US nationals in the SEP through the CDC, wider US government and WHO structures are examined. A most prominent example of this is the relative lack of detailed assessment of the work, negotiations and perspectives provided by major African–American actors like Dr George I. Lythcott, a physician who was the regional lead of the USAID-supported west and central Africa programme, which involved twenty countries and complex negotiations with the governments of each. Female, African–American physicians like Dr Margaret Grigsby, who acted as a CDC consultant in the African campaign, get the odd mention, but their views do not get any sustained historical consideration either. Dr Donald R. Hopkins, who was involved in the SEP and involved in leading current efforts to eradicate guinea worm, appears to be an exception to the trends, possibly because he is an accomplished communicator. See, Donald R. Hopkins, *Princes and Peasants: Smallpox in History* (Chicago: University of Chicago Press, 1983).

5.
See, for example, David, J. Sencer, William H. Foege and James O. Mason, ‘Foreword’, in Ogden, *op. cit.* (note 3), ix–x.

6.
See, ‘Global Health Chronicles: Smallpox’ at: https://www.globalhealthchronicles.org/smallpox-eradication(accessed 1 November 2018).

7.
For example, see William Foege, *House on Fire: The Fight to Eradicate Smallpox* (California: University of California Press, 2012).

8.

Frank Fenner , Smallpox and its Eradication (Geneva: The World Health Organization, 1988).

9.
Donald Ainslie Henderson joined the Smallpox Eradication Unit in the WHO HQ Geneva in November 1966, serving as its chief till 1977. Isao Arita, who had joined WHO as an epidemiologist in 1962, succeeded him, serving between 1977 and 1985. Fenner, *ibid.*, iv.

10.

Isao Arita , The Smallpox Eradication Saga: An Insider’s View (Hyderabad: Orient Blackswan Private Limited, 2010), prologue xvii.

11.

Donald A. Henderson , Smallpox: The Death of a Disease (New York: Prometheus Books, 2009), 61–74.

12.
Arita, *op. cit.* (note 10), 5–6, 18.

13.
Henderson, *op. cit.* (note 11), 58, 65.

14.

Bob H. Reinhardt , The End of a Global Pox: America and the Eradication of Smallpox in the Cold War Era (Chapel Hill: The University of North Carolina Press, 2015), 21, 38.

15.
Reinhardt, *ibid.*, 49.

16.

Erez Manela , ‘A Pox on your Narrative: Writing Disease Control into Cold War History’, Diplomatic History, 34, 2 (2010), 300–303.

17.

Anne-Emanuelle Birn , ‘Small(pox) Success?’, Ciencia & Saude Coletiva, 16, 2 (2011), 598.10.1590/s1413-8123201100020002221340334

18.

Paul Greenough , ‘A Wild and Wondrous Ride: CDC Field Epidemiologists in the East Pakistan Smallpox and Cholera Epidemics of 1958’, Ciencia e Saude Coletiva, 16, 2 (2011), 491–500.2134032410.1590/s1413-81232011000200012

19.
See, for example, Randall Packard, *A History of Global Health: Interventions into the Lives of Other Peoples* (Baltimore: Johns Hopkins University Press, 2016) and Nancy Leys Stepan, *Eradication: Ridding the World of Diseases Forever?* (New York: Cornell University Press, 2011).

20.
Arita, *op. cit.* (note 10), xvii.

21.

Paul Greenough , ‘Intimidation, Coercion and Resistance in the Final Stages of the South Asian Smallpox Eradication Campaign, 1973–75’, Social Science and Medicine, 41, 5 (1995), 633–645.750209710.1016/0277-9536(95)00035-6

22.

Gilberto Hochman , ‘Priority, Invisibility and Eradication: The History of Smallpox and the Brazilian Public Health Agenda’, Medical History, 53, 2 (2009), 240–244.10.1017/s002572730000020xPMC266887919367347

23.

Sanjoy Bhattacharya , Expunging Variola: The Control and Eradication of Smallpox in India, 1947–77 (New Delhi: Orient Longman, 2006).

24.

Sanjoy Bhattacharya , ‘International Health and the Limits of its Global Influence: Bhutan and the Worldwide Smallpox Eradication Programme’, Medical History, 57, 4 (2013), 461–486.2406991310.1017/mdh.2013.63PMC3865968

25.

Vivek Neelakantan , ‘Eradicating Smallpox in Indonesia: The Archipelagic Challenge’, Health History, 12, 1 (2010), 61–87.20973337

26.
Pan American Health Organization, *Document OSP.CE7.W-15*, *Documents of the Seventh Meeting of the Executive Committee, Washington DC, 1949* (Washington DC: Pan American Health Organization, 1949).

27.
World Health Organization, *Official Records of the World Health Organization No. 48* (Geneva: WHO, 1953), 211–15.

28.
World Health Organization, *Official Records of the World Health Organization No. 55* (Geneva: WHO, 1954), 208.

29.

*Ibid.*, 209.

30.
For summaries and text of WHA and EB resolutions about smallpox, see *The Final Report of the Global Commission for the Certification of Smallpox Eradication, Geneva 1979*in collection A33/3 WHO/SE/79.152, Annexe 4, File 844, Box 0001_A, World Health Organization Smallpox Eradication Archives [WHO/SEA].

31.
World Health Organization, *Official Records of the World Health Organization No. 87* (Geneva: WHO, 1958), 508–12.

32.
World Health Organization, *Official Records of the World Health Organization No. 88* (Geneva: WHO, 1958), 7.

33.
Stepan, *op. cit.* (note 19), 194.

34.
WHO, *op. cit.* (note 31), 511.

35.
World Health Organization, ‘*Eradication of Smallpox: Draft Resolution Proposed by the Government of the USSR*’ (Geneva: WHO, 1958) at http://www.who.int/iris/handle/10665/109226(accessed on 20 July 2018).

36.
WHO, *op. cit.* (note 31), 263–72.

37.
‘Smallpox Eradication Report by the Director-General’, 28 April 1959, File 849, Box 1, WHO/SEA.

38.
World Health Organization, *Official Records World Health Organization No. 95* (Geneva: WHO, 1959), 325.

39.
‘Gifts of Smallpox Vaccine EB22.R24’, 14 June 1958, File 1069, Box 599, WHO/SEA.

40.

*Op. cit.* (note 37).

41.
‘Report of the African Conference on Smallpox Eradication WHO/Smallpox/11’, 10 August 1960, File 346, Box 278, WHO/SEA.

42.

*Op. cit.* (note 37).

43.
‘Smallpox Eradication Report by the Director-General’, 19 December 1960, File 849, Box 1, WHO/SEA.

44.

*Ibid*.

45.
For example, see letter from Dr A.C. Saenz, medical officer, WHO HQ, to the Commonwealth Serum Laboratories in Australia, 27 April 1960. File S2-133-1, Box 88, WHO/SEA.

46.
Letter from Dr D. McClean, Lister Institute of Preventive Medicine, London, UK, to Dr A.M. Payne, medical officer, WHO HQ, 5 August 1958, File S2-133-1, Box 88, WHO/SEA.

47.
Letter from Professor A.A. Miles, director, Lister Institute of Preventive Medicine, London, UK, to Dr A.M. Payne, medical officer, WHO HQ, 14 January 1959, File S2-133-1, Box 88, WHO/SEA.

48.
Letter from A.C. Saenz, medical officer, WHO HQ, to Professor A.A. Miles, director, Lister Institute of Preventive Medicine, London, UK, 23 January 1959, File S2-133-1, Box 88, WHO/SEA.

49.
Letter from Professor A.A. Miles, director, Lister Institute of Preventive Medicine, London, UK, to Dr Saenz, medical officer, WHO HQ, 11 February 1959, File S2-133-1, Box 88, WHO/SEA.

50.
For summaries of proposals for pilot projects within WHO regions, see memorandum ‘Smallpox Eradication Programme: Report to the 33rd Executive Board and 17th World Health Assembly’, File 023, Box 2, WHO/SEA.

51.
‘Planning, Organising and Execution of National Smallpox Eradication Campaign in India’, 13 January 1964, File 228, Box 48, WHO/SEA.

52.
Glycerinated vaccines were used everywhere except in Orissa state, where freeze-dried vaccine donated by the Netherlands government, and supplied by the WHO HQ and WHO regional office for South East Asia, was used: *ibid*.

53.
‘The Planning, Organization and Execution of Smallpox Eradication Campaign in Pakistan’, 6 January 1964, File 228, Box 48, WHO/SEA.

54.
World Health Organization, *Official Records World Health Organization No. 103* (Geneva: WHO,1960), 241.

55.
World Health Organization, *Official Records World Health Organization No. 111* (Geneva: WHO,1961), 286.

56.
World Health Organization, *Official Records World Health Organization No. 119* (Geneva: WHO, 1962), 304.

57.
‘Smallpox Eradication Report by the Director-General’, 12 May 1962, File 849, Box 001, WHO/SEA.

58.
WHO, *op. cit.* (note 56), 305.

59.
WHO, *op. cit.* (note 56), 280–7.

60.
WHO, *op. cit.* (note 56), 301–2.

61.
World Health Organization, *Official Records of the World Health Organization No. 128* (Geneva: WHO, 1963), 309.

62.
‘International Certificate of Vaccination or Revaccination Against Smallpox’, 23 December 1963, File 228, Box 048, WHO/SEA.

63.
WHO, *op. cit.* (note 61), 306.

64.
Letter from Dr C. Mani, regional director, WHO regional office for South East Asia, New Delhi, to Ministry of Health of India, New Delhi, 24 December 1964, File S2-133-1, Box 088, WHO/SEA.

65.
‘Smallpox Research’, 29 November 1966, File 023, Box 2, WHO/SEA.

66.
‘Expert Committee on Smallpox: First Report’, File 228, Box 48, WHO/SEA.

67.
Swiss Paediatric Society, ‘Symposium on the Question of Intracutaneous Smallpox Vaccine’, 27 February 1964, File S2-86-1, Box 057, WHO/SEA. This box also contains detailed and wide-ranging correspondence between WHO offices and the society, where SEP strategies are considered.

68.
WHO, *Official Records of The World Health Organization No. 136* (Geneva: WHO, 1964), 346–59.

69.
‘Report on a Visit to the Republic of Mali by a Team of Consultants on the WHO Smallpox Eradication Programme’, *c.*1965, File 935, Box 155_R, WHO/SEA.

70.
See hand-written notes on page 16 of document titled ‘Report on a Visit to Afghanistan by a Team of Consultants on the WHO Smallpox Eradication Programme’, *c.*December 1964, File 935, Box 155_R, WHO/SEA.

71.
‘Extracts from Provisional Minutes EB37/Min/9’, *c.*January 1966, 27, File 1070, Box 599, WHO/SEA.

72.
World Health Organization, *Official Records of World Health Organization No. 144* (Geneva: WHO, 1965), 312–14.

73.
Out of this, US$2.4 million would come from the WHO HQ’s regular budget, and US$0.2 million would be provided by the WHO’s Expanded Programme of Technical Assistance and WHO PAHO. ‘Smallpox Eradication Programme’, 28 March 1966, File 849, Box 1, WHO/SEA.

74.
World Health Organization, *Official Records of the World Health Organization No. 152* (Geneva: WHO, 1966), 258–64, 288–96.

75.

Bichat A. Rodrigues , ‘Smallpox Eradication in the Americas’, PAHO Bulletin, IX, 1 (1975), 53–68. Also see, Fenner *et al.*, *op. cit.* (note 8), 389–91.167890

76.
Stepan, *op. cit.* (note 19), 192–93.

77.

Fred L. Soper , ‘Rehabilitation of the Eradication Concept in Prevention of Communicable Diseases’, Public Health Reports, 80, 10 (1965), 855–869.4953383PMC1919686

78.
Fenner *et al.*, *op. cit.* (note 8), 391.

79.
PAHO, *op. cit.* (note 26).

80.
‘Status of the Smallpox Eradication in America 06/07/1964, annexe II’, File 935, Box 155, WHO/SEA.

81.
‘Status of the Smallpox Eradication in the Americas (CSP 15/17)’, 2 September 1958, File 387, Box 150_R, WHO/SEA.

82.

*Ibid*.

83.

*Ibid*.

84.
Antonio de la Fuente, ‘Informe sobre la Campana de Vacunacion Antivariolica Efectuada Durante el Periodo de Octubre 1950 – Diciembre 1955’, *Boletin de La Oficina Sanitaria Panamericana*, February 1957, File I_B_1, Box 562, WHO/SEA.13412797

85.
‘Status of the Smallpox Eradication in America’, 6 July 1964, File 935, Box 155, WHO/SEA.

86.
‘Status of Smallpox Eradication in the Americas, and Estimated Requirements for the Eradication of Smallpox in the Americas (CSP17/20 Rev.1 Addendum II)’, 24 September 1966, File 935, Box 155_R, WHO/SEA.

87.
‘Status of the Smallpox Eradication in the Americas (CSP 15/17)’, 2 September 1958, File 387, Box 150_R, WHO/SEA.

88.
‘An Alastrim Outbreak in the Gran Sabana (State of Bolivar) Venezuela 1962’, File SMEDOC1-1, Box 414, WHO/SEA.

89.

Harald Frederiksen , Nemesio T. Munoz and Alfredo J. Molina , ‘Smallpox Eradication’, Public Health Reports (1896–1970), 75, 9 (1959), 771–778.PMC192931513824683

90.

*Ibid.*, 771.

91.
Fenner *et al.*, *op. cit.* (note 8), 594.

92.
‘Agreement Between Bolivian Government and PAHO for a Smallpox Eradication Program’, File 446, Box 151R, WHO/SEA.

93.
‘Status of the Smallpox Eradication in America (CD15/22)’, 6 July 1964, File 935, Box 155, WHO/SEA.

94.
‘Status of Smallpox Eradication in the Americas, and Estimated Requirements for the Eradication of Smallpox in the Americas (CSP17/20 Rev.1 Addendum II)’, 24 September 1966, File 935, Box 155_R, WHO/SEA.

95.
‘*Viruela-Paraguay, 1973*’, File 300, Box 151_R, WHO/SEA.

96.
‘Report by the Director-General on Smallpox Eradication, EB37/Min/9:7, Geneva, 1966’, File 1070, Box 599, WHO/SEA.

97.
‘Smallpox in Argentina SMALLPOX/WP/17’, 21 July1967, File 229, Box 048, WHO/SEA.

98.

*Ibid.*, 1.

99.
Hochman, *op. cit.* (note 22), 239.

100.
‘Topic 23: Status of Smallpox Eradication in the Americas’, XV Pan American Sanitary Conference, San Juan, Puerto Rico, Pan American Sanitary Organisation, 1958, File I-B-1, Box 562, WHO/SEA.

101.
‘*A Variola no Brasil*’, File 395, Box 153R, WHO/SEA.

102.
‘Status of the Smallpox Eradication in America’, 6 June 1964, File 936, Box 155, WHO/SEA.

103.
Decree No. 49,974-A, 21 January 1961. A copy of the full document is available in ‘Plan to Eradicate Smallpox in Brazil’, File 939, Box 157 R, WHO/SEA.

104.
‘Report by the Director-General on Smallpox, A13/P&B/14’, 11 April 1960, File 849, Box 001, WHO/SEA.

105.

*Op. cit.* (note 101).

106.
‘Plan to Eradicate Smallpox in Brazil’, File 939, Box 157 R, WHO/SEA.

107.

Ministerio da Saude , 3^°^ Conferencia Nacional de Saude (Brasilia: Ministerio da Saude, 1963), 109–111.

108.
Hochman, *op. cit.* (note 22), 243–44. For detailed assessments of the problems faced by the NCAS, see ‘WHO Memorandum from CMO/IQ to CMQ/VIR, 31 January 1963, WHA16 Document – Smallpox Eradication’, File 026, Box 2, WHO/SEA.

109.
Decree No. 54,366, 1 October 1964. For WHO and WHO PAHO assessments of the situation, see ‘Status of Smallpox Eradication in the Americas, and Estimated Requirements for the Eradication of Smallpox in the Americas. Annex 11’, 24 September 1966. File 935, Box 155, WHO/SEA.

110.
Decree No. 59, 153, 31 August 1966, File 939, Box 157 R, WHO/SEA.

111.

*Op. cit.* (note 106). Also see, ‘Agreement between Brazilian Government and PAHO for a National Smallpox Eradication Programme’, 26 November 1965, File 939, Box 157, WHO/SEA.

112.
Hochman, *op. cit.* (note 22), 245.

113.
Letter from Dr Hector Acuna, medical officer, WHO PAHO, Lima, Peru, to Dr Karel Raska, chief of Communicable Disease, Geneva, 22 August 1966, File IDBRAZIL_0300JKT1, Box 154_R, WHO/SEA.

114.
Letter from Dr Stephen Falkland, interim chief SE/HQ, Geneva, to regional director, WHO AMRO/PAHO, Washington DC, 29 August 1966, File IDBRAZIL_0300JKT1, Box 154_R, WHO/SEA.

115.
Letter from Dr Hector Acuna, medical officer, WHO PAHO, Lima, Peru, to Dr Stephen Falkland, chief SE unit, Geneva, 6 September 1966, File IDBRAZIL_0300JKT1, Box 154_R, WHO/SEA.

116.
Letter from Dr Alfredo Bica, chief Communicable Diseases, WHO AMRO/PAHO, Washington DC to Dr M.G. Candau, WHO DG, Geneva, 8 February 1967, File IDBRAZIL_0300JKT1, Box 154_R, WHO/SEA. Historians have dealt with the important roles played by informal networks and ‘epistemic communities’, created over a period of time, on smallpox vaccine research and production associated to the SEP. See, Steven Palmer and Gilberto Hochman, ‘A Canada–Brazil Network in the Global Eradication of Smallpox’, *Canadian Journal of Public Health*, March/April (2010), 113–14, 118. However, as these scholars acknowledge, the connections between these networks and SEP developed mainly post-intensification of the SEP.

117.
‘Status of Smallpox Eradication in the Americas, and Estimated Requirements for the Eradication of Smallpox in the Americas. Addendum II’, 24 September 1966, 3, File 935, Box 155, WHO/SEA.

118.
‘Status of Smallpox Eradication in the Americas and Estimated Requirements for the Eradication of Smallpox in the Americas, Washington DC, 1966’, in PAHO Document CSP17/20, Rev. 1, English, File 394, Box 152_R, WHO/SEA.

119.
‘Status of Smallpox Eradication in the Americas, and Estimated Requirements for the Eradication of Smallpox in the Americas (CSP17/20 Rev.1 Addendum II)’, 24 September 1966, File 935, Box 155_R, WHO/SEA.

120.
See two drafts of ‘Agreement between the Argentinian Government and PAHO for a Smallpox Eradication Programme’, *c.*March 1967, File 446 Box 151_R, WHO/SEA.

121.
‘Agreement for a Smallpox Vaccination Program and Epidemiologic Surveillance in Colombia’, 2 May 1967, File 446, Box 151R, WHO/SEA.

122.
‘Operational Plan for Peru as Part of the Continental Smallpox Eradication Program’, File 446, Box 151R, WHO/SEA.

123.
‘*Acuerdo Para un Programa de Vacunacion Y Vigilancia Epidemiologica en Viruela en el Ecuador*’, File 446, Box 151_R, WHO/SEA.

124.
‘Report by the Director-General on Smallpox Eradication’, 20 April 1967, File 849, Box 001, WHO/SEA. Strikingly, the WHO HQ got more involved in vaccine production and quality testing in Latin America once the SEP’s intensification had received WHA support. This empowered the WHO HQ to work with regional offices and member states to create new scientific exchanges and work towards strengthening existing connections. An important article analyses the biases and hostility of Robert J. Wilson, a Canadian scientist involved in a WHO-supported programme intended to improve Brazilian smallpox vaccine production, towards national laboratories and production facilities. See, Steven Palmer, Gilberto Hochman and Danieli Arbex, ‘Smallpox Eradication, Laboratory Visits, and a Touch of Tourism: Travel Notes of a Canadian Scientist in Brazil’, *Historia, Ciencias, Saude – Manguinhos*, 17, 3, (2010), 777–90.10.1590/s0104-5970201000030001221461539

125.

*Smallpox Eradication – Report of a WHO Scientific Group: World Health Organization Technical Report Series, No. 393* (Geneva: WHO, 1968), 25, Official Publications Room, University of Cambridge Library, Cambridge, UK.

126.
‘Smallpox Eradication Programme A19/P&B/2’, 28 March 1966, File 849, Box 1, WHO/SEA.

127.
‘The Smallpox Eradication Programme – Preliminary Outlook’, *c.*1967, File 27, Box 3, WHO/SEA.

128.
‘Provisional Summary of the 12th Meeting, Committee on Programme and Budget, A21/P&B/SR/12’, 20 May 1968, File 849, Box 1, WHO/SEA.

129.
Letter from Dr F. Soliman, chief, Public Health Administration (PHA), WHO HQ, Geneva, to Dr S. Falkland, medical officer, SE Unit, WHO HQ Geneva, 19 November 1966, File 23, Box 2, WHO/SEA.

130.
‘Questions Which Might be Asked by the Board in Connection to item 2.6 of Provisional agenda of EB39’, *c.*1966, File 23, Box 2, WHO/SEA.

131.
‘Smallpox Eradication EB31’, *c.*1963, File 23, Box 2, WHO/SEA.

132.
‘Conclusions and Recommendations Arising out of the Technical Discussions held at the 17th Session of the Regional Committee for South-East Asia’, New Delhi, 28 September 1964, File 849, Box 1, WHO/SEA.

133.
Letter from Dr I. Arita, medical officer, SE Unit, WHO HQ, Geneva, to Dr P.M. Kaul, assistant director-general, WHO HQ Geneva, 22 December 1965, File 23, Box 2, WHO/SEA.

134.
Letter from Dr M. Candau, WHO DG, WHO HQ, Geneva, to all WHO regional directors, 15 June 1966, File 27, Box 3, WHO/SEA.

135.
‘Smallpox Eradication Programme EB41/12’, 11 December 1967, File 1069, Box 599.

136.
See, for example, personal letter from Dr N. Maltseva, medical officer, WHO SEARO, New Delhi, to D.A. Henderson, Chief, Smallpox Eradication, WHO HQ, Geneva, 27 June 1967, File 416, Box 193, WHO/SEA.

137.
Memorandum from regional director, WHO SEARO, New Delhi, to D.A. Henderson, chief, Smallpox Eradication, WHO HQ, Geneva, 5 January 1968, File 416, Box 193, WHO/SEA.

138.
Memorandum from D.A. Henderson, chief, Smallpox Eradication, WHO HQ, Geneva, to regional director, WHO SEARO, New Delhi, 5 February 1968, File 416, Box 193, WHO/SEA.

139.
See, for example Ogden, *op. cit.* (note 3). For a good write-up on how histories of the SEP influenced choices and decisions in subsequent eradication programmes, see Ciro de Quadros, ‘The last challenge: the Horn of Africa’, in Sanjoy Bhattacharya and Sharon Messenger (eds), *The Global Eradication of Smallpox* (Hyderabad: Orient BlackSwan, 2010), 103–5.

